# Salix Nigra, &c.

**Published:** 1889-02

**Authors:** F. T. Paine

**Affiliations:** Comanche, Tex.


### SALIX NIGRA, THE NEW SEXUAL SEDATIVE.

BY F. T. PAINE, M. D., COMANCHE, TEX.

In April, 1885, I had the pleasure of presenting this new medicine to the notice of the profession, in a paper read before the Texas State Medical Association, at its session held at Houston. This paper has been very generally reproduced in the medical journals of this country, and, being entirely new, has attracted no little attention both in and out of the profession, as my extensive correspondence throughout the Union for the last three years demonstrates.

That there should have been some failures and disappointments is not very surprising, in the use of a remedy so entirely new and for troubles so very extensive and varied.

But the disappointments are chiefly due to an omission on the part of the writer of the original paper.

In that paper a hope was expressed that the new medicine would become invaluable in the treatment of masturbation and spermatorrhœa, omitting to state that it is only in the initial stages of these troubles that benefit may be expected, and that it is only prophylactic.

The diseases incident to, and consequent upon masturbation and excessive venery are both more numerous, more troublesome, and more difficult to get clear of than the plagues of Egypt. And I now never attack them except with the aid of electricity.

As to the value of salix nigra as a true sexual sedative, I had then no doubt, and now after three years of additional experience, I have the positive confirmation.

In my original paper a case of dysmenorrhœa of 16 years' standing was reported as cured, I had reason then for believing that it was also a case of nyphomania, though the woman refused then, and again recently, to answer interrogations on the subject. She would only use the prescription until relieved of pain, and is now a subject of diseased ovary.

In a few weeks I called on this lady, when she returned the half-emptied bottle of salix nigra, saying she had no use for the balsam, nor any use for——, her husband, only to furnish grub and make fires, and that she never knew what health was before. On careful investigation I found the enlargement, hyperæmia and the hyperæsthesia of the ovary all gone. The vulva, vagina, uterus and ovaries were perfectly natural, as far as a digital examination could reveal. The cure of the vaginismus was due to electricity; the reduction in size, the hyperamia and hyperæsthesia of ovary, to salix nigra.

April, 1888, a maiden lady of about 50 years came under treatment for troubles, which I considered reflex, from ovarian trouble. On examination I found the ovaries much enlarged and exceedingly sensitive to the touch. Ordered fluid extract nigra ʒi, three times daily. In ten days the ovaries were very much reduced in size and sensitiveness, and the patient very much improved in health. But on the approach of another period in the catamenial cycle some of the same troubles returned, but she was so well satisfied the improvement was due to the salix that she immediately ordered another bottle.

These cases prove that the action of the medicine is directly and specifically upon the testicular organs of the sexes, reducing hyperæmia and hyperæsthesia. This condition of hyperæmia and hyperæsthesia is agreed by common consent to obtain on the approach and during the œstrual of animals and catamenial epoch in the human female, and the recuction of this state is the philosophy of the use of the salix nigra.

In pursuance of this thought, how much of the so-called criminal life of women is due to a pathological state of the ovaries, which is amenable to treatment? But she may demand treatment for anything, rather than erotism. She may fly from yellow fever, but her erotism follows her and is relentless. I firmly believe she will find in the salix a "balm in Gilead."

One man was reported who confessed to six copulations nightly, and who never was satisfied sexually, but after ten days' use of the salix could not copulate once a week. Another of the cases reported cured came five years later to be cured of the effects of masturbation. While the drug suppresses the sexual passions, it will not cure the evils already produced by sexual excesses.

I had an opportunity in April last of applying the crucial test. A lady aged 23, applied for treatment, whose history, mixed up with diagnosis and treatment of her family physician, obscured the case so as to add greatly to the difficulty of a correct diagnosis. She was married three years without a conception, had been told she had abscess of the uterus, and that tumors blocked the vulvo-vaginal canal.

I found vaginisimus and proposed separating her from her husband until cured, which being positively refused, I found nymphomania existing as the cause of the refusal. The vulvo-vaginal hyperæsthesia precluded the possibility of a digital investigation—even the presence of the thermometer was intolerable. Yet, so great was the sexual impulse that she could not and would not forego the coitus. She said the orgasm was continuous; during intercourse her pleasure never ceased. That if she had not married when she did, she would have lost her virtue, and sought illicit gratification. In her first intercourse, the nuptial couch was rendered a pool of blood (perhaps an explanation of some other reported cases of ruptured vagina in a first intercourse), yet she made no complaint on the occasion. Contrary to my expectations, it required thirteen days to cure the vaginismus, using electricity one hour each day. (I reported a case to the Texas State Medical Association, at Austin April, 1886, cured in one sitting of thirty minutes). The vaginismus cured, I freely examined the ovaries, and found them hyperæmic, enlarged, and very sensitive, hyperæsthetic, as in the case first reported in original paper, in which the woman refused to answer as to sexual feelings.

For the nyphomania, I ordered the fluid extract of salix nigra (P., D. & Co.'s) ʒ j, t. i. d.—*Med Age.*

